# Predictive Factors of Mortality in Burn Patients

**DOI:** 10.5812/traumamon.14480

**Published:** 2014-01-25

**Authors:** Shahram Fazeli, Reza Karami-Matin, Neda Kakaei, Samira Pourghorban, Roya Safari-Faramani, Bahare Safari-Faramani

**Affiliations:** 1 Department of Surgery, Kermanshah University of Medical Sciences, Kermanshah, IR Iran; 2 Department of Biostatistics and Epidemiology, Kermanshah University of Medical Sciences, Kermanshah, IR Iran; 3 Department of Statistics, Razi University of Kermanshah, Kermanshah, IR Iran

**Keywords:** Risk Factors, Burns, Mortality

## Abstract

**Background:**

Burn injuries impose a considerable burden on healthcare systems in Iran. It is among the top ten causes of mortality and a main cause of disability.

**Objectives:**

This study aimed to examine factors influencing mortality in burn patients admitted to the main educational tertiary referral hospital in Kermanshah.

**Patients and Methods:**

All patients admitted to the Imam Khomeini Hospital (from March 2011 to March 2012), due to thermal burn injuries were included in the study. We applied multiple logistic regressions to identify risk and protective factors of mortality. Also we calculated lethal area fifty percent (LA50), as an aggregate index for hospital quality.

**Results:**

During the study period, 540 burn patients were admitted. Male to female ratio was 1.12:1. Twenty three percent of the patients were less than 15 years-old. Median of age was 25 years (Inter Quartile Range, 16 - 37). Overall, probability of death was 25.8%. Lethal area fifty percent (LA50) was 50.82 (CI 95%: 47.76 - 54.48). In the final model, after adjustment of sex, age, total body surface area (TBSA), cause of burn and it’s severity, female gender (P < 0.05), age ≥ 60 years (in comparison with age less than 15 years, P < 0.05) and larger burn size (P < 0.0001) were identified as the main risk factors of death in these patients.

**Conclusions:**

Findings showed that the main risk factors of death were female gender, burn size and old age. Directing more attention to these vulnerable patients is required to reduce mortality and improve patient survival.

## 1. Background

Annually, near 200000 people die due to burn injuries around the world (1). Burn injuries are among the public health concerns across the globe particularly in developing countries. The highest mortality rates of burn injuries are reported form southeastern Asia followed by the east Mediterranean region (1, 2). In Iran burn injuries impose a considerable burden on the healthcare system. It is the eighth cause of years of life lost (YLL) and the thirteenth cause of death and disability-adjusted life years (DALY) (3). Given the significant burden of this problem in Iran, describing epidemiologic characteristics and identifying potential factors affecting the mortality of burn patients may help identify patients at risk of death. High total body surface area (TBSA) burns, old age, female gender, and inhalation injury are identified as mortality risk factors (4-6). Although there are plenty of studies on the epidemiology of burns in Iran, sufficient studies on mortality risk factors have not been performed (7). Multivariable models to identify mortality risk factors are among the priorities of research in this area (7). 

## 2. Objectives

In this study we aimed to examine factors influencing mortality in burn patients while adjusting for other factors by multivariable models.

## 3. Patients and Methods

Kermanshah province with a population about two million people and 13 administrative districts, is located in the western part of Iran. Imam Khomeini Hospital, affiliated to Kermanshah University of Medical Sciences, a tertiary referral teaching hospital. The burn ward of this hospital admitted patients from all districts of the province as well as patients referring from adjacent provinces. During the study period, a registry system was established to collect required data. A trained person was responsible for filling the forms by asking the victims or their attendants the designed questions. All patients admitted to the hospital due to thermal burn injuries between March 20, 2011, and March 21, 2012, were included in the study. Patients were considered in an open-cohort study and were followed from admission to discharge. Demographic data were collected by interviewing patients or their attendants. Data on injury were collected by reviewing the patient files. Total body surface area (TBSA) burns were calculated by using the rule of nines or the Lund-Browder diagram. This project was approved by Research Committee of Kermanshah University of Medical Sciences. We applied multiple logistic regressions to identify mortality risk and predicting factors. Crude and adjusted odds ratios (OR) with 95% confidence intervals are reported. For univariable and multiple logistic regressions, statistical significance level was set at P < 0.05. Also we calculated lethal area fifty percentage (LA50), an aggregate index for hospital quality, by applying probit analysis (LA50 is the TBSA burn where the probability of death for the patient is 50 percent). 

## 4. Results

During the study 540 burn patients were admitted. Data on final outcome for 145 patients were missed. We included these patients in descriptive analysis and excluded them for analytical statistical analysis by complete case analysis in multiple logistic regression. 

**Figure 1.**
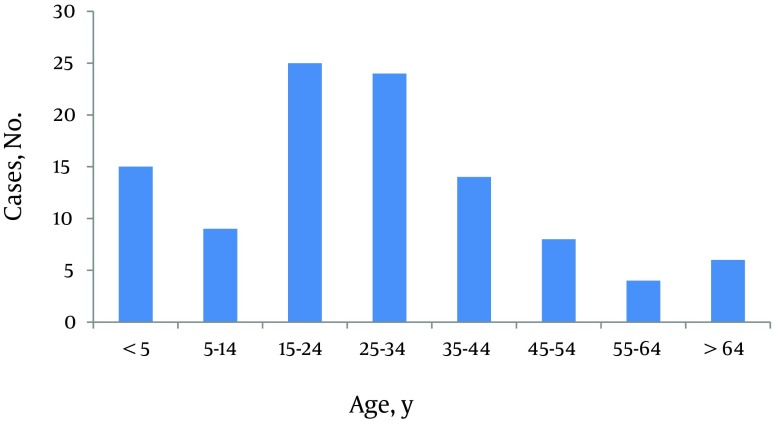
Age Distribution of the Patients Referred to the Burn Center

**Figure 2.**
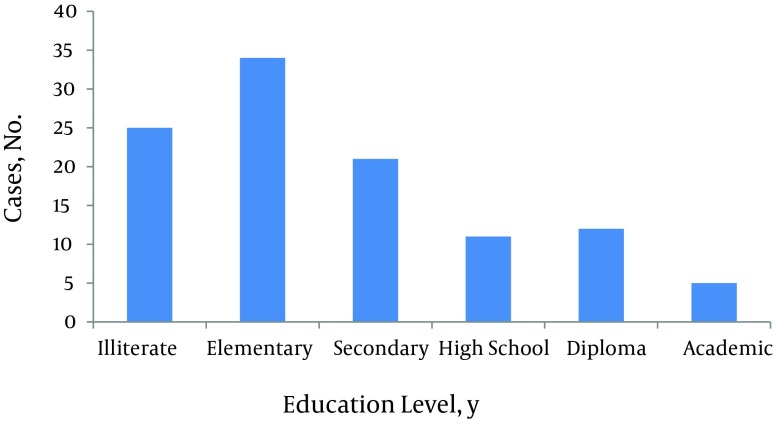
Distribution of the Burn Patients According to the Education Level

**Figure 3.**
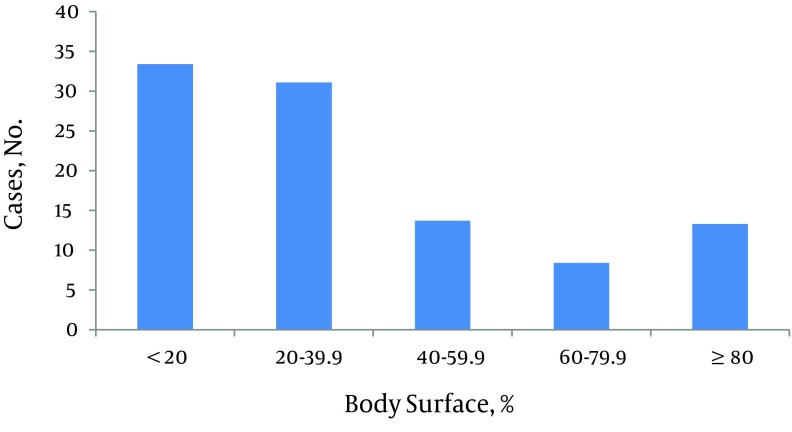
Distribution of patients’ Total Body Surface Area Burned

**Figure 4.**
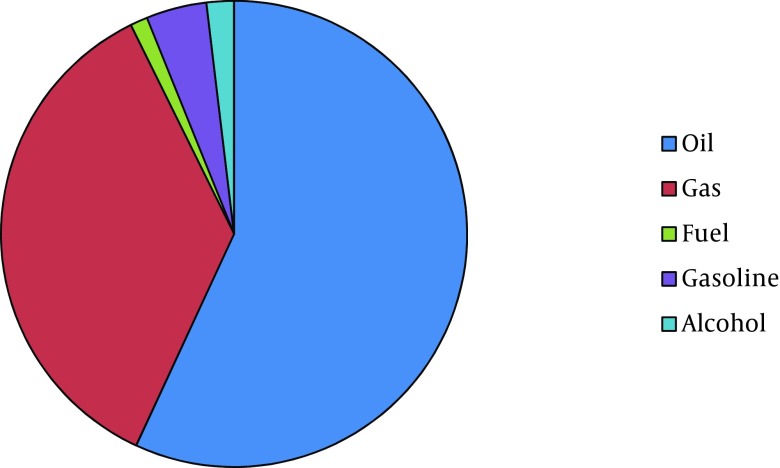
Distribution of Substances Used by the Victims that Led to Burns

**Figure 5.**
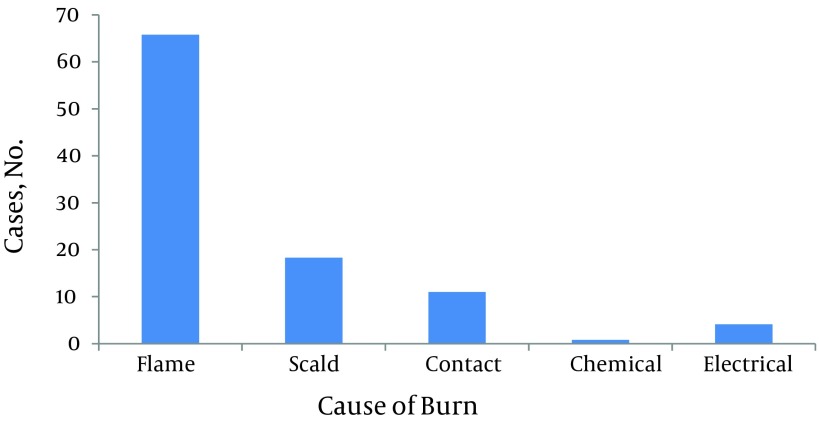
Distribution of Causes of Burn Among the Patients

Male to female ratio was 1.12:1. Of total, two hundred eighty five patients were male. Age range was from 9 months to 89 years-old. Median of age was 25 (IQR 16 - 37). Twenty three percent of the patients were less than 15 years-old. Among children less than fifteen, 63% were less than five years-old and also male gender was dominant in this age group. But in patients aged 15 to 24 years-old, females were over represented. Most of the patients were from Kermanshah Province (94.6%) and about six percent were referred from adjacent provinces. The TBSA of burns ranged from 1 to 100%. The median was 27.5 percent (IQR 16-53). About one third of patients (33.4%) had TBSA burn less than twenty percent and 21.7 % had TBSA burn of 60 percent or more. There were 18 cases with 100% TBSA burn. The most common cause of burn was flames (65.8%) followed by scalding (18.3%). Causes of burns in different age groups were varied. In patients younger than 15 years-old, the most common cause was scalding (43.6%) and in patients aged 15 to 60 years and aged ≥ 60 years, it was flames (80.2 and 70.6 percent, respectively). Burns from substance in the cases of flame injury for more than half of the victims was kerosene (Figures 1-5). More than 60 percent of cases were accidental, 30.1 percent were suicidal, and the remaining related to occupational injuries. Mean age in accidental cases was 25.43 years-old (range from 9 month to 89 years-old), in occupational cases was 34.34 years-old (range: 19 - 69 years-old), and in suicide cases was 29.48 years-old with a range from 11 to 84 years (P < 0.005). The median of TBSA burned in these three groups was as follows: In accidental cases it was 20 percent, in occupational cases it was 12.75%, and in suicidal cases it was 62.5%. These differences were statistically significant (P < 0.0001). Mean length of hospital stay in accidental cases was 6.87 days (SD: 6.50). In occupational cases it was 6.82 days (SD: 6.94) and in suicidal cases 11.03 days (SD: 12.49). These differences were also statistically significant (P < 0.0001). In addition, male to female ratio in accidental cases was 1.88:1. In suicidal cases it was 0.165:1. Forty one out of forty two occupational cases were male.

Overall probability of death was 25.8%. Probability of death in accidental cases was 9.9 percent, in occupational cases was 5.9%, and it was 63.6% in suicidal cases. LA50 was 50.82 (CI 95%: 47.76 - 54.48) for all patients. Among cases with 100% TBSA burn, data on final outcome was missed for four of them, and eight of them died during the first 24 hours of admission. Three patients died during the first 48 hours after admission, two patients survived within six days and one of them died after 23 days. In univariable logistic models, females were nearly four times more vulnerable to death than males (crude OR: 3.88, P < 0.0001). In multivariable model this relationship remained significant (adjusted OR was 9.02, P < 0.05). Patients who were aged ≥ 60 years-old were more than 12 times at risk in comparison with children younger than 15 years-old (crude OR: 12.08, P < 0.0001). Also patients aged between 15 and 60 years-old were around seven times more at risk of death (crude OR: 6.84, P < 0.0001). In the multivariable model, the adjusted OR of death in patients aged ≥ 60 years-old in comparison with children less than 15 years was 19.74 (P < 0.05). There were no statistically significant differences between patients aged 15 to 60 years and children less than 15 years-old after adjusting for other variables (P = 0.179). With an increase of one percent of TBSA, probability of death from burns increased by 17 percent, and by 24 percent when adjusted for other variables. Crude OR of death for flame burns was 16.69 (5.95 - 46.81), when compared with burns caused by other substances, in the multiple logistic regression model this relationship was no longer statistically significant (P = 0.131) while the OR was 5.33. Odds ratio for death was 16.69 (5.95 - 46.81) times more in suicidal cases in comparison to accidental and occupational in the univariable model but in multivariable model this relationship was not significant (P = 0.089)(Table 1).

**Table 1.** Probability of Death, Crude and Adjusted Odds Ratio of Death Among the Subgroups

Variable	Death Risk, %	Crude Odds Ratio, [ 95% Confidence Interval]	P-value	Adjusted Odds Ratio, [95% Confidence Interval]	P-value
**Sex**					
Male	14.2	1			1
Female	39.1	3.88 [2.38- 6.31]	< 0.0001	9.02 [1.35 - 60.01]	0.02
**Age groups, y**					
< 15	5.7	1		1	
15 - 60	29.4	6.84 [2.67 - 17.52]	< 0.0001	0.24 [0.03 - 2.93]	0.18
> 60	42.4	12.08 [3.88 - 37.65]	< 0.0001	19.74 [1.37 - 284.41]	0.03
**TBSA^a^**		1.17 [1.13 - 1.21]	< 0.0001	1.24 [1.15 - 1.33]	< 0.0001
**Cause of burn**					
Flame	35.7	16.69 [5.95 - 46.81]	< 0.0001	5.33 [0.61 - 1.29]	0.13
Other causes	3.2	1		1	
**Intention **					
Accidental or Occupational	9.4	1		1	
Self-immolation	63.6	16.77 [9.67 - 29.09]	< 0.0001	0.19 [0.03 - 1.29]	0.09

^a^Abbreviations: TBSA, total body surface area

## 5. Discussion

The primary goal of this study was to identify mortality risk and predicting factors in burn injuries. In this study, the risk of death among burn patients was 25.8% and LA50 was 50.8%, which meant that it was estimated that half of the patients with nearly 51 percent TBSA burned would die. Findings showed female gender, age over 60, and larger burn size were the main risk factors of death. Probability of death in the present study was higher than other studies (8-13). This may be because of high proportion of severe burn injuries in Iran confirmed by the distribution of TBSA. In most previous studies with low mortality, probability mean of TBSA burned was less than 15 (8-14), while in this study, TBSA burned for more than half of the patients was around 30. Thus, severity of burn injuries will be a cause of higher probability of death. Muller et al. and Sharma et al. reported that the probability of death in females was higher than males even after adjusting for TBSA (4, 11) and the result of our study confirmed this. Although in some studies this relationship was found in univariable analysis (15, 16), in some cases there was no relationship (17, 18). Flame burns was one of the mortality risk factors in univariable analysis but after adjusting for TBSA this relationship was no longer significant. In other words, as found in other studies (16), the relationship was confounded by the burn size as TBSA was a main risk factor for death. There was no significant relationship between intention of injury and the final outcome. Suicidal cases had larger burn sizes than the accidental cases (P < 0.0001). In multivariable model, when considering burn size, there was no relationship between intention and death. Similar to other studies, the most important risk factor of death was burn size (4, 17). The most vulnerable age group in this study was patients aged 60 years-old and older. This finding was consistent with the results of other studies (4, 17). Self-immolation is one of the most common methods of suicide, in Iran. In this study, like previous studies in Iran, a relatively high proportion of burn cases were due to suicidal behaviors (16, 19, 20). Sex ratio on suicidal cases was different from accidental cases and most of the time women were the prominent victims. In suicidal cases the, number of females was six times greater than males. This pattern was similar to other studies that were carried out in Iran (21-23). However, this pattern was different in developed countries, where most of the time suicide by burning was equally distributed between males and females and sometimes it was higher among men (24-27). Also the proportion of suicidal cases was far less than Iran (28-30). But in most parts of Iran and other developing countries the main victims were women (31-33). The pattern of burn causes in age groups was different. Studies reported that most of the time the cause of burn was scalding in children and flames in older people (7, 12, 14). Also In this study, the most common cause of burns among children younger than 15 years was scaldijg and among older patients was flames. The main strength of this study was applying multivariable models to investigate mortality risk factors. As the main risk factors were female gender, burn size, and old age, it is necessary to emphasize that these patients are more vulnerable to death and need more attention as a strategy to reduce mortality and improve patient survival after burn injury. Also strengthening the infrastructure is needed to treat these patients. There were limitations in our study. One of the main limitations was lack of data on immediate cause of death and on inhalation injury as one of the main risk factors of death in burn patients. Also data on degree of burns was not available and we could not adjust its effect. These shortcomings must be considered in future studies. Recording and managing the data on burns is one of the main research priorities in Iran. There is no formal registry system for this purpose. Uniform evaluation of burn centers, data on death and causes of death in burn patients, and also data on therapeutic procedures will help policy makers decide on the most appropriate services. It also helps evaluate the main risk factors of death and to identify vulnerable groups. Therefore, it is highly recommended to establish an integrated registry system across the country to reduce mortality and improve patient survival.

## References

[1] Mathers C, Fat DM, Boerma J (2008). The global burden of disease: 2004 update..

[2] Ahuja RB, Bhattacharya S (2004). Burns in the developing world and burn disasters.. BMJ..

[3] Naghavi M, Abolhassani F, Pourmalek F, Lakeh MM, Jafari N, Vaseghi S (2009). The burden of disease and injury in Iran 2003.. Pop Health Metr..

[4] Muller MJ, Pegg SP, Rule MR (2001). Determinants of death following burn injury.. Br J Surg..

[5] Grunwald TB, Garner WL (2008). Acute burns.. Plast Reconstr Surg..

[6] O'Keefe GE, Hunt JL, Purdue GF (2001). An evaluation of risk factors for mortality after burn trauma and the identification of gender-dependent differences in outcomes.. J Am Coll Surg..

[7] Sadeghi-Bazargani H, Mohammadi R (2012). Epidemiology of burns in Iran during the last decade (2000-2010): review of literature and methodological considerations.. Burns..

[8] Jie X, Baoren C (2003). Mortality rates among 5321 patients with burns admitted to a burn unit in China: 1980-1998.. Burns..

[9] da Silva PN, Amarante J, Costa-Ferreira A, Silva A, Reis J (2003). Burn patients in Portugal: analysis of 14,797 cases during 1993-1999.. Burns..

[10] Chien WC, Pai L, Lin CC, Chen HC (2003). Epidemiology of hospitalized burns patients in Taiwan.. Burns..

[11] Sharma PN, Bang RL, Ghoneim IE, Bang S, Sharma P, Ebrahim MK (2005). Predicting factors influencing the fatal outcome of burns in Kuwait.. Burns..

[12] Ho WS, Ying SY (2001). An epidemiological study of 1063 hospitalized burn patients in a tertiary burns centre in Hong Kong.. Burns..

[13] Pegg SP (2005). Burn epidemiology in the Brisbane and Queensland area.. Burns..

[14] Maghsoudi H, Samnia N (2005). Etiology and outcome of pediatric burns in Tabriz, Iran.. Burns..

[15] Anlatici R, Ozerdem OR, Dalay C, Kesiktas E, Acarturk S, Seydaoglu G (2002). A retrospective analysis of 1083 Turkish patients with serious burns. Part 2: burn care, survival and mortality.. Burns..

[16] Groohi B, Alaghehbandan R, Lari AR (2002). Analysis of 1089 burn patients in province of Kurdistan, Iran.. Burns..

[17] Macedo JL, Santos JB (2007). Predictive factors of mortality in burn patients.. Rev Inst Med Trop Sao Paulo..

[18] Kobayashi K, Ikeda H, Higuchi R, Nozaki M, Yamamoto Y, Urabe M (2005). Epidemiological and outcome characteristics of major burns in Tokyo.. Burns..

[19] Saadat M (2005). Epidemiology and mortality of hospitalized burn patients in Kohkiluye va Boyerahmad province (Iran): 2002-2004.. Burns..

[20] Mohammadi AA, Danesh N, Sabet B, Jalaeian H, Mohammadi MK (2008). Self-burning: a common and tragic way of suicide in Fars Province, Iran.. Iran J Med Sci..

[21] Mohammadi AA, Danesh N, Sabet B, Amini M, Jalaeian H (2008). Self-inflicted burn injuries in southwest Iran.. J Burn Care Res..

[22] Rastegar Lari A, Alaghehbandan R (2003). Epidemiological study of self-inflicted burns in Tehran, Iran.. J Burn Care Rehabil..

[23] Lari AR, Joghataei MT, Adli YR, Zadeh YA, Alaghehbandan R (2007). Epidemiology of suicide by burns in the province of Isfahan, Iran.. J Burn Care Res..

[24] Pham TN, King JR, Palmieri TL, Greenhalgh DG (2003). Predisposing factors for self-inflicted burns.. J Burn Care Rehabil..

[25] Rothschild MA, Raatschen HJ, Schneider V (2001). Suicide by self-immolation in Berlin from 1990 to 2000.. Forensic Sci Int..

[26] Seoighe DM, Conroy F, Hennessy G, Meagher P, Eadie P (2011). Self-inflicted burns in the Irish National Burns Unit.. Burns..

[27] Nakae H, Zheng YJ, Wada H, Tajimi K, Endo S (2003). Characteristics of self-immolation attempts in Akita Prefecture, Japan.. Burns..

[28] Palmu R, Isometsa E, Suominen K, Vuola J, Leppavuori A, Lonnqvist J (2004). Self-inflicted burns: an eight year retrospective study in Finland.. Burns..

[29] Tsati E, Iconomou T, Tzivaridou D, Keramidas E, Papadopoulos S, Tsoutsos D (2005). Self-inflicted burns in Athens, Greece: a six-year retrospective study.. J Burn Care Rehabil..

[30] Theodorou P, Phan VT, Weinand C, Maegele M, Maurer CA, Perbix W (2011). Suicide by burning: epidemiological and clinical profiles.. Ann Plast Surg..

[31] Ahmadi A, Mohammadi R, Stavrinos D, Almasi A, Schwebel DC (2008). Self-immolation in Iran.. J Burn Care Res..

[32] Laloe V, Ganesan M (2002). Self-immolation a common suicidal behaviour in eastern Sri Lanka.. Burns..

[33] Mabrouk AR, Mahmod Omar AN, Massoud K, Magdy Sherif M, El Sayed N (1999). Suicide by burns: a tragic end.. Burns..

